# Cenobamate in Real‐Word Scenario: Results on Efficacy, Side Effects, and Retention Rate in a Single Center Retrospective Study

**DOI:** 10.1002/brb3.70567

**Published:** 2025-05-18

**Authors:** Mostafa Badr, Christoph Helmstaedter, Susanna Moskau‐Hartmann, Jan Pukropski, Juri‐Alexander Witt, Theodor Rüber, Karmele Olaciregui Dague, Tobias Baumgartner, Michael Rademacher, Rainer Surges, Randi von Wrede

**Affiliations:** ^1^ Department of Epileptology University Hospital Bonn Bonn Germany

**Keywords:** cenobamate, efficacy, real‐world data, retention rate, side effects

## Abstract

**Background:**

Pharmacoresistance imposes a high burden on people with epilepsy (PWE). Recently authorized cenobamate (CNB) offers new hope with high efficacy reported in phase III and early real‐world studies. Here, we present data from a reasonably sized monocentric cohort, complementing the knowledge derived from clinical practice.

**Methods:**

We retrospectively analyzed medical records of all PWE treated with CNB from market entry to July 31, 2023.

**Results:**

After an average of 1.1 years, follow‐up data were available for 262 out of 280 PWE, who received at least one dose of CNB. The average CNB dose was 183 ± 98 mg/d, with a mean number of anti‐seizure medications (ASM) of 2.9 ± 1 per patient. A total of 36% of the patients showed ≥ 50% reduction in seizure frequency (10.7% were seizure‐free), whilst 12.3% reported increased seizure frequency. Seizure freedom was associated with concomitant perampanel or GABA receptor modulators. No predictors of treatment response were found. Side effects were reported by 38%, alertness issues being most prevalent (19%). The retention rate amounted to 72% and was associated with response status, dose of CNB, side effects, and age at CNB introduction. Among those who discontinued CNB, 55% experienced side effects and 89% showed no meaningful seizure reduction. Regarding co‐medication, the withdrawal of lamotrigine, brivaracetam, clobazam, or lacosamide was associated with higher rates of non‐response.

**Conclusions:**

In this large cohort of 262 PWE, CNB proved very efficient with a high retention rate over one year. Co‐medication with perampanel or GABA receptor modulators was linked to seizure‐freedom. The overall positive impression of CNB is further supported.

## Graphical abstract

This monocentric study in a large cohort demonstrated high efficacy and a favorable retention rate of CNB after an average of one year of treatment. These results support CNB as an effective long‐term therapeutic option for patients with DRE.

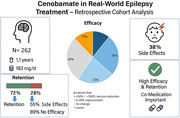

## 1 Introduction

Epilepsy remains a challenging neurological disorder affecting millions of people worldwide and is a significant global health issue, impacting 0.5%–1% of the general population over their lifetime (Marson et al. 2007; Van Den Broek and Beghi 2004). Despite the availability of various anti‐seizure medication (ASM) targeting different mechanisms of action, including modulation of γ‐aminobutyric acid‐ergic (GABAergic) activity, sodium and calcium ion channels, and others, approximately one‐third of epilepsy patients still do not achieve seizure freedom (Dwivedi et al. 2017; Kwan et al. 2010). According to the International League Against Epilepsy (ILAE) commission, failure of two appropriately chosen ASMs to control seizures defines drug‐resistant‐epilepsy (DRE) (Téllez‐Zenteno et al. 2014). Patients with DRE experience an increased risk of morbidity, mortality, and reduced quality of life, as well as significant socioeconomic constraints (Mula and Cock 2015; Tomson et al. 2005). In case that the first ASM proves ineffective, the subsequent ASM offers an additional 11.6% chance of seizure freedom. Only a minority of patients with DRE achieve optimal seizure control with successive ASMs (Chen et al. 2018). Additionally, for some patients, epilepsy surgery is a promising treatment option, but may not be suitable for all patients (Engel et al. 2012; Wiebe et al. 2001). Last but not least, neurostimulation techniques such as vagus nerve stimulation, deep brain stimulation, and focal cortex stimulation are emerging treatments for DRE (Schulze‐Bonhage et al. 2023). Despite the aforementioned options, there is still a need for new, effective ASMs for better seizure control.

Cenobamate (YKP3089, CNB) is a novel ASM that has been shown to have a broad spectrum of anti‐seizure activity in rodent models of epilepsy (Bialer et al. 2013). In November 2019, the United States Food and Drug Administration (FDA) granted approval for CNB to manage drug‐resistant focal‐onset seizures (Commissioner of the FDA 2020), followed by the European Medicine Agency in March 2021. Recent studies showed that CNB significantly reduced focal seizures in patients with DRE (Brigo and Lattanzi 2024; Krauss et al. 2020; Rissardo and Fornari Caprara 2023). The mechanism of action of CNB remains incompletely understood, it has been demonstrated to act on voltage‐gated sodium channels as well as a positive allosteric modulator of GABA‐A receptors, differently from benzodiazepines binding sites (Roberti et al. 2021). CNB can inhibit the activity of cytochrome P450 (CYP) 2C19, while simultaneously inducing the activity of CYP3A4 and 2B6. Consequently, it can potentially interact with other ASMs, which may demand dose adjustments for lamotrigine, carbamazepine, and clobazam, among others (Zaccara et al. 2021). Notable side effects, including somnolence, dizziness, fatigue, and diplopia may occur with CNB, they are dose‐related, most of them manifest early in the titration phase, and they resolve over time (Schuetz et al. 2022). Nevertheless, the most pertinent safety concerns currently relate to the potential for severe skin reactions (which may be mitigated through a gradual dose titration) and QT shortening (Roberti et al. 2021). Although a dose effect of CNB has been postulated, suggesting greater efficacy with higher CNB doses (Peña‐Ceballos et al. 2023), an Italian expert opinion paper reports a significant effect in many patients already at relatively low doses (Villani et al. 2022). Additionally, two other studies indicate that dosage in not related to efficacy or side effects (Novitskaya et al. 2024; Villanueva et al. 2023). Furthermore, it has been demonstrated in certain studies that the efficacy of CNB may be better in patients with severe epilepsy (Peña‐Ceballos et al. 2023).

The present monocentric retrospective study reports real‐world outcomes on clinical efficacy, safety, and potential drug‐by‐drug interactions in a cohort of patients with epilepsy (PWE) who received CNB and investigates predictors for the above‐mentioned outcomes.

## 2 Material and Methods

### 2.1 Study Design

A retrospective search of the local ASM registry, approved by the Ethics Commission of the Medical Faculty at the University of Bonn (approval number 130/19), was conducted to identify all patients who initiated CNB therapy between its market entry and July 31, 2023. The inclusion criteria comprised adult patients (≥ 18 years) with a diagnosis of focal epilepsy in accordance with the International League Against Epilepsy (ILAE) guidelines. Patients were excluded if they were under 18 years of age or had a diagnosis of generalized epilepsy.

Overall, a total of 309 patients fulfilled the inclusion criteria. Among these patients, 306 (99%) had DRE before initiating the CNB therapy. Follow‐up data until January 31, 2024, were available for 262 of the 309 patients. A total of 29 patients were lost to follow‐up and 18 patients discontinued CNB prior to revisiting. Clinical data, including demographics, age at onset of epilepsy, education level, mental disability, and lifetime ASMs were extracted from the electronic patient files and cross‐checked by another physician. Seizure frequency was gathered on the basis of self‐assessment and categorized into categories according to the Revised Seizure‐based Outcome Classification System (Duke) with Analysis of Relationship to health‐related quality of life (HRQOL) (Vickrey et al. 1995) into three groups: seizure free, ≤10 seizures a year, and >10 seizures a year according to Vickrey (Vickrey et al. 1995). Since most patients were in the last group, latter was additionally divided into three subgroups. The first subgroup, comprising patients with less than one seizure per week, was designated as “mild to moderate”. The second subgroup, designated as “severe” included patients with one or more seizures per week but less than one seizure per day. The third subgroup, which was defined as patients with “very severe” epilepsy, included individuals with one seizure per day or more. Side effects, number of concomitant ASMs, accumulated total drug load according to the defined daily doses (DDD), and retention rates were documented at follow‐up. The DDD shows the “assumed average maintenance dose per day” for drugs used in different medical areas (Hollingworth and Kairuz 2021). In this particular instance, our focus is on ASMs. The actual daily dose of a specific ASM is related to the respective DDD by calculating the ratio actual daily dose/DDD. The total or cumulative DDD is the sum of these ratios for all AMSs within an individual drug regimen.

### 2.2 Outcome Parameters and Statistical Analysis

The study outcomes were efficacy, retention, and side effects. Efficacy was calculated by the relative change in seizure frequency from baseline to follow‐up; responders were defined as reductions in seizure rates of 50% and higher. Retention rate was defined as all the patients which still on CNB therapy at the last visit. Side effects were retrospectively extracted from patients’ electronic health records, which contained documentation by healthcare providers during routine clinical visits. A qualitative analysis of all reported treatment‐related side effects was conducted, and side effects were categorized as “none”, or related to “somnolence”, “physiological”, “mood”, “physical”, “cognition” related symptoms by three independent raters to ensure consistency and objectivity.

Descriptive statistics were employed to assess demographic characteristics and efficacy. Quantitative continuous variables were presented as mean ± standard deviation (SD) or range, while nominal variables were expressed as *n* (%). The *t*‐test was employed for continuous variables, while the chi‐square test was used for categorical variables to compare differences.

Regression analysis was used to identify potential predictors of treatment response, retention, and side effects. The chi‐square test was also utilized to determine the potential relationship between co‐medication and seizure freedom, treatment retention, or side effects. Any instances of missing data were duly noted. The statistical analysis was conducted using IBM SPSS Statistics (v28) software.

## 3 Results

### 3.1 Demographic Data

The cohort under study comprised 309 PWE, of which 280 received CNB add‐on (47.9% female, 52.1% male) with a mean age of 40.9 ± 14.3 years. A total of 29 patients (9.3%) were lost to follow‐up. Almost the entire study cohort (99%) exhibited DRE, with a median of nine different ASMs previously used (range: 1–23). Only one patient had only one ASM before initiating CNB treatment. A total of 120 patients (42.9%) exhibited psychiatric comorbidities, with 71 patients (25.4%) reporting a history of mental retardation. Education information was available for 250 patients, with 106 (42.4%) having more than 9 years of education, while 144 patients (57.6%) had less than 10 years of education or more. None of the 280 patients was seizure‐free at the time of CNB introduction. A total of 18 patients (6.5%) had ten or fewer seizures per year, while 261 patients (93.5%) had more than 10 seizures per year (1 missing). At baseline, 81 patients (29%) were classified as belonging to the “mild to moderate epilepsy” subgroup, 130 patients (46.6%) were identified in the “severe epilepsy” subgroup, and 68 patients (24.4%) were classified as belonging to the “very severe epilepsy” subgroup. Consequently, 71% of the patients included in the study exhibited severe to very severe refractory epilepsy. A comprehensive overview of the demographic and clinical characteristics of the study group can be found in Table 1. The total number of concomitant ASMs taken prior to the initiation of CNB therapy was **2.9 ± 1** per patient, in comparison to 3.1 ± 1.3 at the retention phase. The total DDD at baseline amounted from 4 ± 1.7, in comparison to 4.0 ± 1.8 at the retention phase. The mean dose of CNB was 183 ± 98 mg/d. The average observation time was 1.1 years (0.03–2.7, SD 0.7) for the 262 patients who remained in the final analysis Table 2.

**TABLE 1:** Demographic data.

Sex	*N* = 280	Female Male	*N* = 134 *N* = 146	47.9% 52.1%
**Age at onset of epilepsy** (years)	*N* = 276 Missing = 4		Mean 15.8, SD 14.2	
**Age at start of CNB** (years)	*N* = 280		Mean 40.9, SD 14.3	
**Psychiatric comorbidity**	*N* = 280		Yes, *N* = 120	42.9%
**Mental retardation**	*N* = 280		Yes, *N* = 71	25.4%
**Education level**	*N* = 250 Missing = 30	(<10 yrs)	*N* = 144	57.6%
		(≥10 yrs)	*N* = 106	42.4%
**Lifetime ASMs**	*N* = 276 Missing = 4		Median 9 Range 1–23, Mean 9.4 SD 4.1	
**Baseline seizure frequency**	*N* = 279 Missing = 1	Seizure free	0	0%
		(≤10/yr)	*N* = 18	6.5%
		(>10/yr)	*N* = 261	93.5%
**Severity**	*N* = 279 Missing = 1	<1/week (mild to moderate)	*N* = 81	29%
		≥1/week,<1/day (severe)	*N* = 130	46.6%
		≥1/day (very severe)	*N* = 68	24.4%

*Note*: Values are presented as number (%), mean (range; SD), unless otherwise specified.

**TABLE 2:** DDD and Number of concomitant of ASMs.

	*N*	Min	Max	Mean	SD
**DDD at start of CNB**	280	0.6	11.4	4,0	1.7
**Number of concomitant ASMs at start with CNB** **(without CNB)**	280	1	5	2.9	1
**DDD at phase of retention**	262	0.03	10.6	4.0	1.8
**Number of concomitant ASMs at phase of retention (without CNB)**	262	0	6	3.1	1.3
**Average dose of CNB (mg) at phase of retention**	262	6.25	500	183	98

*Note*: Values are presented as number, mean (min‐max; SD), unless otherwise specified.

### 3.2 Efficacy, Retention Rate, and Side Effects

**FIGURE 1:**
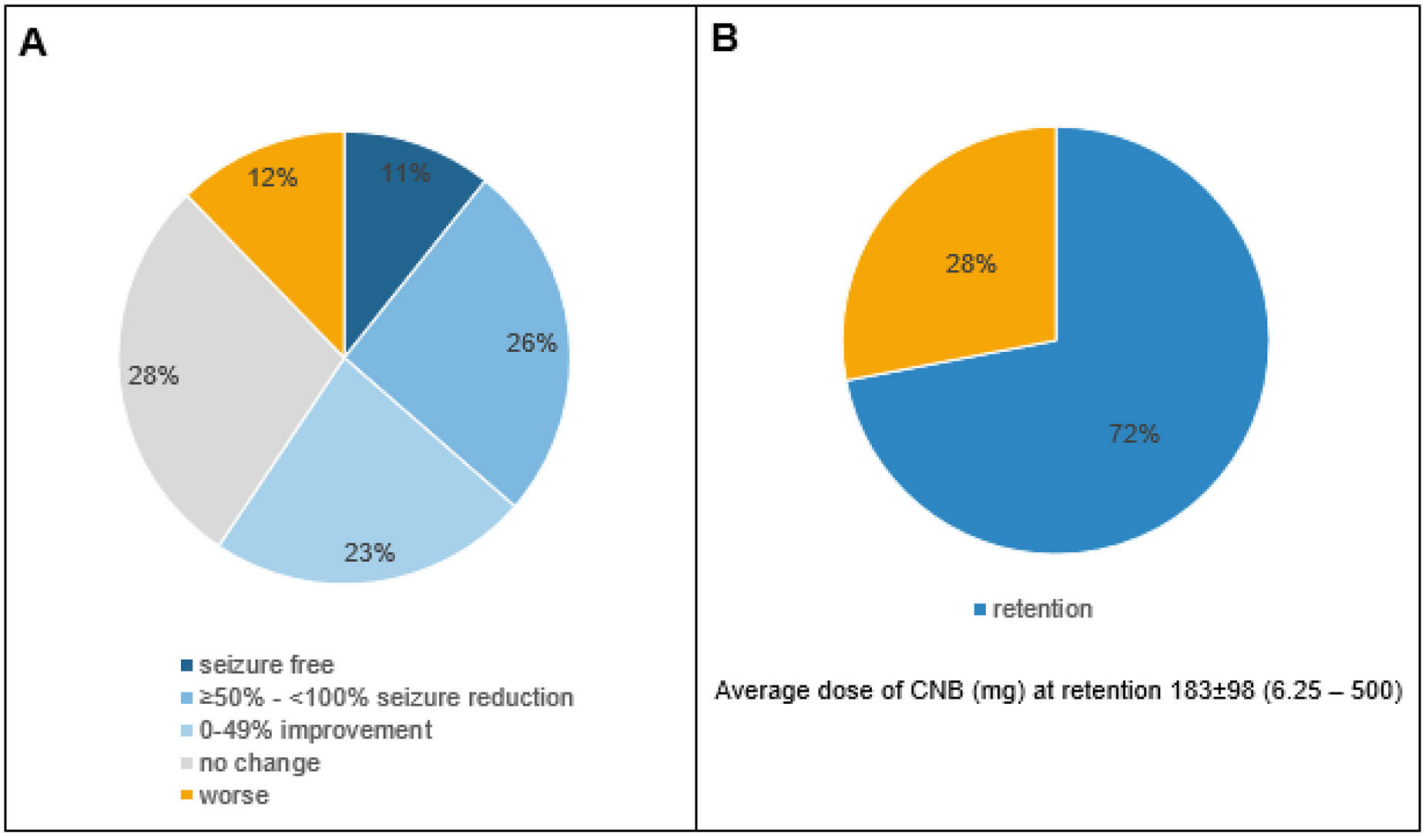
Efficacy and retention of CNB.

A total of 36.4 % of PWE exhibited a positive response to treatment (10.7% were seizure‐free), while 12.3% experienced an increase in seizure frequency (Figure 1). Neither the dosage nor the age, gender, number of previously prescribed ASMs, epilepsy severity, mental impairment, or the presence of psychiatric comorbidities were found to be significantly related to treatment response.

**FIGURE 2:**
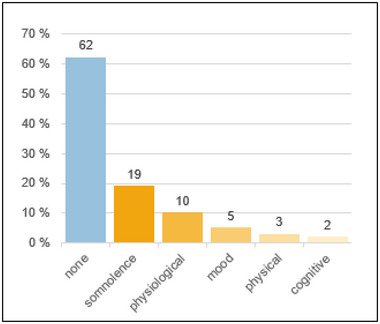
Side effects of CNB.

Side effects were reported by 38% of patients. Regarding the entire cohort, the most common side effects were somnolence, followed by physiological problems. Mood, physical, and cognitive problems were less common (Figure 2).

The retention rate was 72%. In those who discontinued CNB, 55% experienced side effects, and non‐response was observed in 89%.

### 3.3 Potential Predictors and Impact of Co‐Mediation

The ordinal regression analysis for treatment response did not provide any significant result, when considering the predictor variables gender, age at onset of epilepsy, psychiatric comorbidities, mental status, education level, number of previously used ASMs, DDD, dose of CNB, dose of CNB and number of concomitant ASMs. The logistic regression analysis for side effects displays only significance regarding preexisting psychiatric comorbidity (*p* = 0.03). The potential predictor variables gender, age at onset of epilepsy, mental status, response status, dose of CNB, education level, age at CNB introduction, number of concomitant ASMs, number of previously used ASMs, DDD, seizure frequency score, and disease severity (Vickrey et al. 1995) did not enter the prediction model. According to logistic regression analysis retention was determined by response status, dose of CNB, education level, side effects, age at CNB introduction. Lower education level, higher age at CNB introduction and higher CNB doses were associated with higher retention rates (see Table 3). The variables gender, age at onset of epilepsy, psychiatric comorbidities, mental status, number of concomitant ASMs, number of previously used ASMs, DDD, seizure frequency score, and disease severity, did not turn out to be predictive.

**TABLE 3:** Logistic regression model for predictors.

	Model	Variables	*B*	SE	df	Wald	Significance
Side effects	Chi‐square 4.596; df 1, Sig 0.32 Predicted correct percentage 60.6	Psychiatric comorbidity	0.590	0.276	1	4.566	0.032
Retention	Chi‐square 58.607; df 15, Sig < 0.01 Predicted correct percentage 77.4	Responder	2.159	0.44	1	24.102	< 0.001
		Dose of CNB	0.006	0.002	1	10.034	0.002
		Education level	−1.056	0.358	1	8.679	0.003
		Side effects	−0.874	0.345	1	6.401	0.011
		Age at CNB introduction	0.026	0.012	1	4.365	0.037

**FIGURE 3:**
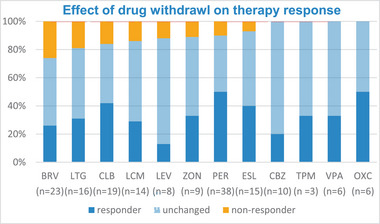
Percentage of withdrawal of concomitant ASMs and its effect.

Finally, the chi‐squared test was used to determine the potential influence of co‐medication on seizure freedom, treatment retention, and side effects. Regarding seizure freedom, the results indicate a positive influence of co‐medication with perampanel (chi2 = 4.9, df 1, *p* = 0.025) and GABA modulators (chi2 = 3.6, df 1, *p* = 0.044). Only 6% of patients achieved seizure freedom with co‐medication of levetiracetam and brivaracetam, in contrast to 18% who achieved seizure freedom without these drugs (chi2 = 9.142, df 1, *p* = 0.003, = 0.02). Co‐administration of potassium, calcium channel blockers, or sodium channel blockers with CNB did not have a significant effect on seizure freedom, responder rates, retention, or side effects. In case of efficiency, drugs were withdrawn within the observation interval. The investigation of the impact of the withdrawal of concomitant ASMS on CNB treatment response demonstrated a higher incidence of non‐response when withdrawing brivaracetam (26%), lamotrigine (19%), clobazam (16%), and lacosamide (14%) (Figure 3).

## 4 Discussion

The findings from our study provide real‐world insights into the demographics, clinical characteristics, and treatment outcomes of patients with pharmacoresistant epilepsy receiving CNB as an add‐on therapy, thereby complementing the regulatory studies on CNB. The reported cohort primarily consists of patients with severe DRE with a high burden of prior treatment failures, as seen in smaller cohorts before (Novitskaya et al. 2024; Rodríguez‐Uranga et al. 2024), underscoring the refractory nature of the epilepsy in this cohort and the critical need for effective alternative treatments.

Our analysis of 262 patients with DRE revealed a response rate of 36%, with 11% achieving seizure freedom. While, CNB appears an effective treatment, the response rate is lower than expected from pivotal or previous first real‐world data, which ranged between 50% and 85% (Brigo and Lattanzi 2024; Krauss et al. 2020; Novitskaya et al. 2024; Steinhoff et al. 2024; Winter et al. 2024; Chung et al. 2020). The discrepancy in seizure freedom rates compared to pivotal studies can easily be attributed to the shorter observation periods of the latter. With longer observation intervals, patients may cease CNB therapy due to non‐titration side effects or due to a worsening of seizure outcomes. Variations observed in other real‐world scenarios suggest center specific issues and suggest the need for pooling data from multiple centers controlling for different healthcare systems.

The retention rate of 72% over an average interval of 1.1 years in our cohort reflects a very good efficacy, tolerability, and adherence to the therapy. This retention rate is consistent with recent findings (80%) (Steinhoff et al. 2024) and it is higher compared to other third‐generation ASMs, for example, 67% for perampanel or 65% for lacosamide or 52% for brivaracetam (Steinhoff et al. 2024). The COMPARE study reveals a further decline in retention rates for some ASMs after 12 months (Roberti et al. 2024). Thus, longer term follow‐up will be necessary to reveal the final durability of the efficacy of CNB.

A total of 38% of the patients of our cohort reported side effects associated with CNB. This parallels previously reported numbers (Novitskaya et al. 2024; Steinhoff et al. 2024), but the rate is significantly lower than in the pivotal studies (Krauss et al. 2020; Chung et al. 2020), as to be expected, given the well‐defined clear procedures employed in randomized controlled trials (RCTs) and as evidenced by similar findings for other ASMs (Rohracher et al. 2018; Krauss et al. 2012). In alignment with the preceding studies, somnolence was the most prevalent side effects (Krauss et al. 2020; Steinhoff et al. 2024; Chung et al. 2020). Discontinuation due to side effects was in line with recent studies (Krauss et al. 2020; Novitskaya et al. 2024).

An interesting observation is that seizure freedom was more likely with concomitant perampanel treatment, potentially due to different mechanisms of action. However, first experiences with higher risk of increased side effects under perampanel‐cenobamate co‐medication were reported (Steinhoff et al. 2024). Similarly, a higher chance for seizure freedom was observed with concomitant use of GABA modulators, possibly indirectly due to increased serum concentrations of clobazam and its active metabolite, N‐desmethylclobazam (Elakkary et al. 2023). In contrast, concomitant treatment with levetiracetam or brivaracetam was associated with a lower chance of seizure freedom. Albeit, the results of the statistical analysis have to be interpreted with caution, due to relatively small number of the analyzed group.

From clinical point of view in DRE, seizure freedom is optimal, nevertheless relevant reduction in seizure frequency measured by response rate is a crucial goal in this group. Good medical practice and the proposed treatment recommendations demand simplification or at least stabilization of the number of concomitant ASMs and the DDD (Carreño et al. 2024; Smith et al. 2022). Our findings, including similar numbers of concomitant ASMs and the DDD before the introduction of CNB and in the retention phase, indicate adherence to these recommendations. We found higher rates of non‐response, when the co‐medications with brivaracetam, lamotrigine, clobazam or lacosamide are withdrawn in the course of the therapy, which should be taken into account when deciding which medication to discontinue. Further prospective studies should address this important issue, as it has significance for medical decision making.

As for all other concomitant medications, no significant correlations regarding side effects or treatment retention were identified, suggesting that CNB may act independently, indicating further distinctive mechanism of action, which could become crucial when tapering or withdrawing co‐medication once seizures are successfully controlled.

Apart from concomitant ASM we did not identify any predictors for efficacy in general in our cohort. In particular, there was no notable enhanced efficacy of CNB in patients with severe epilepsy (69% of our cohort) as reported by others (Peña‐Ceballos et al. 2023; Beltrán‐Corbellini et al. 2023). Furthermore, our findings do not support a dosage‐dependent effect of CNB, consistent with the other studies (Novitskaya et al. 2024; Villanueva et al. 2023).

As expected, positive treatment response and absence of side effects were positive predictors for retention. Our study showed that higher retention rates can be expected particularly in older subjects, those who tolerate higher doses and those with less education. However, this demands vice versa that for younger and more educated, presumable more critical, subjects more diligent and careful titrations schemes should be considered.

The only predictor found for side effects was pre‐existing psychiatric comorbidity, reflecting the disease immanent higher propensity to complain side effects, as already shown by other ASMs (Kanner et al. 2021). However, it is important to consider the potential drug interactions and influence of other medications commonly prescribed to patients with psychiatric comorbidities, such as antidepressants (Helmstaedter and Witt 2012; Helmstaedter et al. 2024). This should be addressed in future studies.

The strengths of this study are the real‐world setting in a tertiary epilepsy center, the large patient cohort and the comparably long follow‐up period. The retrospective design and incomplete follow‐up data for some patients represent limitations that should be considered.

In conclusion, our findings indicate that CNB seems to be an effective add‐on therapy per se but even for patients with severe DRE. Our study findings in regard to the co‐medication support the suggestion that CNB has a mode of action which is not shared by other ASMs. Future studies should directly focus on the question of which ASM have additive effects and which co‐medication in polytherapy can be tapered or withdrawn without jeopardizing the positive effect of CNB, and addressing the urgent question of CNB monotherapy.

## Author Contributions

**Mostafa Badr**: conceptualization, data curation, investigation, methodology, visualization, writing–original draft, writing–review and editing, formal analysis, software, validation. **Christoph Helmstaedter**: methodology, software, validation, formal analysis, writing–review and editing, visualization, supervision. **Susanna Moskau‐Hartmann**: data curation, investigation, validation. **Jan Pukropski**: data curation, investigation, validation, writing–review and editing. **Juri‐Alexander Witt**: formal analysis, validation, writing–review and editing. **Theodor Rüber**: validation, investigation, data curation. **Karmele Olaciregui Dague**: validation, data curation, investigation. **Tobias Baumgartner**: data curation, investigation, validation. **Michael Rademacher**: data curation, investigation, validation. **Rainer Surges**: supervision, writing–review and editing, formal analysis, conceptualization. **Randi von Wrede**: conceptualization, methodology, software, data curation, investigation, validation, formal analysis, writing–review and editing, supervision.

### Peer Review

The peer review history for this article is available at https://publons.com/publon/10.1002/brb3.70567.

## Data Availability

The data that support the findings of this study are available on request from the corresponding author. The data are not publicly available due to privacy or ethical restrictions.
